# Effects of body movement on yaw motion in bipedal running lizard by dynamic simulation

**DOI:** 10.1371/journal.pone.0243798

**Published:** 2020-12-31

**Authors:** Jeongryul Kim, Hongmin Kim, Jaeheung Park, Hwa Soo Kim, TaeWon Seo

**Affiliations:** 1 Center for Healthcare Robotics, Korea Institute of Science and Technology, Seoul, South Korea; 2 School of Mechanical and Aerospace Engineering, Seoul National University, Seoul, South Korea; 3 Department of Intelligence and Information, Seoul National University, Seoul, South Korea; 4 Advanced Institutes of Convergence Technology (AICT), Suwon, Gyeonggi-do, South Korea; 5 Department of Mechanical System Engineering, Kyonggi University, Suwon-si, South Korea; 6 School of Mechanical Engineering, Hanyang University, Seoul, South Korea; University of Münster, GERMANY

## Abstract

Lizards run quickly and stably in a bipedal gait, with their bodies exhibiting a lateral S-shaped undulation. We investigate the relationship between a lizard’s bipedal running and its body movement with the help of a dynamic simulation. In this study, a dynamic theoretical model of lizard is assumed as a three-link consisting of an anterior and posterior bodies, and a tail, with morphometrics based on *Callisaurus draconoides*. When a lizard runs straight in a stable bipedal gait, its pelvic rotation is periodically synchronized with its gait. This study shows that the S-shaped body undulation with the yaw motion is generated by minimizing the square of joint torque. Furthermore, we performed the biomechanical simulation to figure out the relationship between the lizard’s lateral body undulation and the bipedal running locomotion. In the biomechanical simulation, all joint torques significantly vary by the waist and tail’ motions at the same locomotion. Besides, when the waist and tail joint angles increase, the stride length and duration of the model also increase, and the stride frequency decreases at the same running speed. It means that the lizard’s undulatory body movements increase its stride and help it run faster. In this study, we found the benefits of the lizard’s undulatory body movement and figured out the relationship between the body movement and the locomotion by analyzing the dynamics. In the future works, we will analyze body movements under different environments with various simulators.

## 1. Introduction

The running patterns of vertebrates vary depending on the species. The development of these running patterns is known to be an evolutionary result of adapting to the environment. Biologists use several methods, such as comparative approaches, to study the principles of observed running patterns. In the field of biomechanics, the dynamics analysis technique is used to verify the principles of running patterns by simulation. In this study, we explain the principle of the running pattern of a fast bipedal running lizard. More specifically, we investigate the effect of lateral body undulation on bipedal running at high speed by simulating a theoretical mechanical model of a running lizard.

A lizard can run in a straight line with a very stable posture even at a high speed. While running, it has been observed that a lizard bends its body and tail in the horizontal plane, which is referred to as a lateral body undulation, in the same manner as snakes and fish. Because this movement is quite large and dynamic, we hypothesize that such a body movement plays an important role in the bipedal lizard’s locomotion.

In previous studies, the lateral undulation of lizards has been observed. Ritter observed the unique S-shaped lateral body undulation of a lizard with its various running speeds [1]. Whether such a lateral undulation is a result of active movement or passive movement has also been investigated. Ritter analyzed the electromyography and kinematic data of the epaxial muscle of a running lizard and concluded that the lizard actively bends its waist to stabilize its trunk [2,3]. Similarly, by analyzing the electromyography of the epaxial muscle, Bennett *et al*. confirmed that at a low speed, the epaxial muscle resists the body’s torsional force, whereas at a high speed, the muscle is used to bend the waist [4]. These studies showed that the lateral body undulation is not a result of a passive external force, but of active internal moments. However, these studies did not explain the actual purpose of such a lateral body undulation or its effect on locomotion.

The effect of a lizard’s tail was also studied. Jusufi *et al*. and Libby *et al*. studied the balancing effect of the tail when a lizard free-falls or jumps [5,6]. Gillis *et al*. showed that after tail loss, a lizard could no longer control its body when it jumps [7]. Kim *et al*. improved the motion stability of a six-legged water-running robot in the yaw and pitch direction using a two degrees-of-freedom (DOF) active tail [8]. MSU Tailbot can control body angle for a safe landing with swing tail in the air [9]. Such studies have focused on the tail’s role in the lizards’ pitch and roll. On the other hand, TAYLRoACH can change its running direction as 90° turns at a constant rotational speed of 360°/s using a 1 DOF active tail in the horizontal plane [10]. *Salamandra Robotica* controlled by the Central Pattern Generator (CPG) is similar to that of a real salamander in the horizontal plane [11]. These robots studied the yaw movement of the body, but the effect of the periodic lateral bending of the body during running was not investigated.

The contributions of this paper are to 1) find the benefits of the lateral undulations of the lizard body during bipedal running, and 2) confirm the relationship between the lizard’s undulatory body movement and the bipedal running locomotion. The target of this study, *Callisaurus draconoides*, is a creature that can run very fast compared to its size, and the body of the lizard dynamically moves in the yaw direction. We analyze the effect of the lateral body undulation of a steady-state bipedal running lizard on its yaw motion via a dynamic theoretical simulation. Besides, we simulate the effect of the undulatory body motion on bipedal running locomotion through a dynamic simulation using a lizard biomechanical model with the legs. The biomechanical model derived in this study has 33 DOFs, based on the morphometrics of *Callisaurus draconoides*. The extensive simulations verify that the undulatory body movements dominantly affects its bipedal running locomotion.

## 2. Analysis for the benefit of the lizard’s undulatory body movement

When a lizard runs straightly with a bipedal gait, its posterior body swings symmetrically and periodically in the horizontal plane. Furthermore, the lateral undulations of a lizard generate a periodical S-shaped movement. When the lizard’s running is stable, the angle between the trunk and the tail is very close to zero [12]. It means that if the lizard ideally balances the body, the undulatory body movement will occur only in the horizontal plane. Therefore, we assumed an ideal situation that the lizard was stably balanced, and modeled the lizard’s undulatory body movement only in the horizontal plane. In addition, we studied the relationship between the theoretical model’s running performance and the movement of the lizard body using the optimization method and assuming the data obtained from the previous studies.

### A. Three-link theoretical model of a lizard

**Fig 1:**
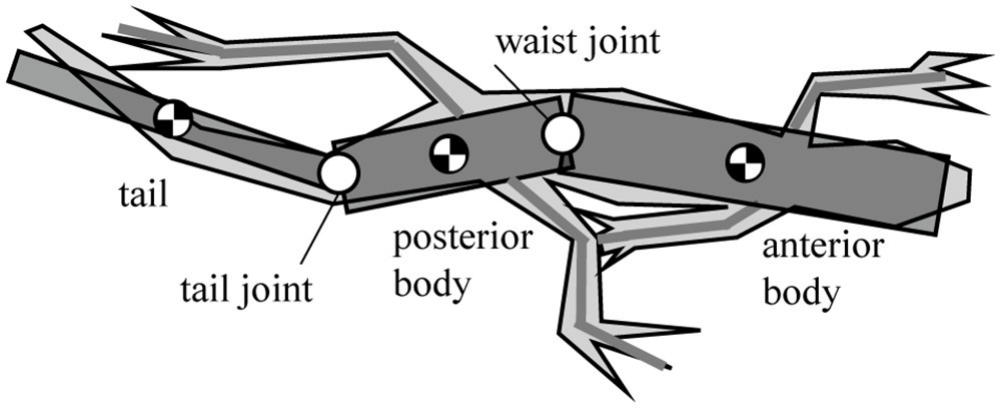
Three-link theoretical model of a lizard, *Callisaurus draconoides*, in the horizontal plane. The moment of inertia for the segment of the body is assumed as a homogenous cylinder. Since the mass percentages of the forelegs and the hind limbs are low relative to the total body mass, the leg motion’s effect is assumed to be negligible.

After observing the *Callisaurus draconoides*, a theoretical model comprising a three-link system with an anterior and posterior bodies, and a tail is devised as shown in Fig 1. The joints are located such that each link can rotate in the horizontal plane relative to its neighboring links. Animal muscles have both rigid and flexible properties so that when generating force actively, they are rigid but when absorbing forces like springs, they become passive and flexible. This study modeled a lizard’s joint as the rotating joint to mimic the active muscle and extract joint torque. The forelegs are attached to the anterior body and the hind legs to the posterior body. The moment of inertia for the segment of the body is calculated by assuming the segment as homogenous cylinder. The morphometrics of *Callisaurus draconoides* obtained from the real measured data in [13]. For such lizards as *Anolis sagrei* and *Anolis carolinensis*, which are similar to the *Callisaurus draconoides* and run with a bipedal gait, Legreneur *et al*. found that the mass percentages of the forelegs relative to the total body mass were 2.05% and 1.8%, and those for the hind limbs were 6.2% and 4.25%, respectively [14]. Thus, the effect of the leg motion is assumed to be negligible, and the leg masses are included in the anterior and posterior bodies. The morphometrics of *Callisaurus draconoides* used for the three-link theoretical model are summarized in Table 1, and the details of the derivation of the three-link theoretical model are presented in Appendix A1.

**Table 1:** Morphometrics of *Callisaurus draconoides* used for the three-link theoretical model.

*Callisaurus draconoides*	
Total length (mm)	192
Anterior body length (mm)	66
Posterior body length (mm)	42
Tail length (mm)	84
Anterior body width (mm)	20
Posterior body width (mm)	20
Tail width (mm)	6
Total mass (g)	10
Anterior body mass (g) (with fore leg added)	4
Posterior body mass (g) (with hind leg added)	4
Tail mass (g)	2

### B. Kinematic analysis of a lizard’s bipedal running

**Fig 2:**
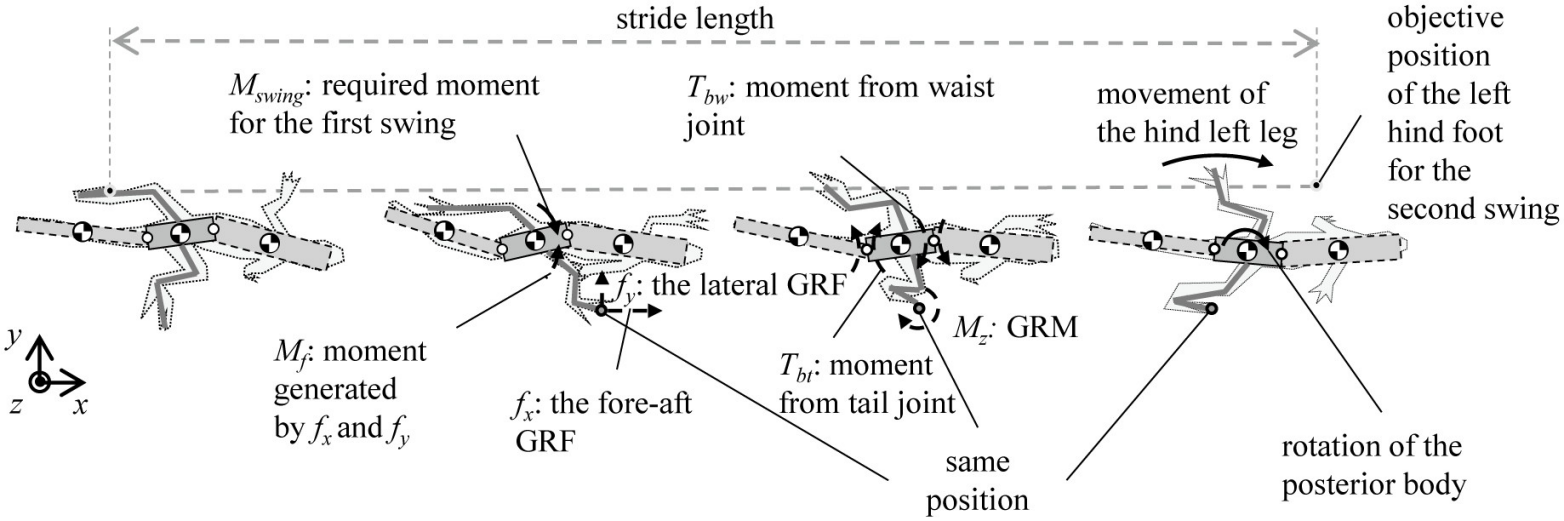
Moments and forces periodically acting on the lizard in a period in the horizontal plane. When a lizard runs, its legs exert a force on the ground with every stride, a fore-aft ground reaction force GRFx (*f*_*x*_), lateral force GRFy (*f*_*y*_), and GRM are produced at its feet. Also, the body torques (*T*_*bw*_, *T*_*bt*_) are generated by the bodies’ joint movement. A lizard controls these forces, moments, and torques to run forward by repeating the gait.

Fig 2 shows the forces and moments acting on each body segment of the lizard when it runs straightly with a bipedal gait. The roles of the ground reaction force (GRF), ground reaction moment (GRM), and lateral undulation of the waist and tail of a lizard must be uncovered for the analysis. When a lizard runs, its legs exert a force on the ground with every stride, and as a result, a fore-aft ground reaction force GRFx (*f*_*x*_) and lateral force GRFy (*f*_*y*_) are produced at its feet. Besides, GRFz (*f*_*z*_) occurs in the height direction of the lizard’s foot. GRFz is closely related to the roll and pitch motion of the lizard body. However, the body of the lizard is assumed to have relatively little movement in the roll and pitch directions compared to the yaw direction from the previous studies [12,15]. Therefore, the effect of GRFz on the lizard’s undulatory body movement was judged to be small, and the force analysis of the lizard was performed in the horizontal plane, excluding GRFz, as shown in Fig 2. If we consider that the right leg swings first while the left leg is in the stance stage, the GRF produces both a translational force and a moment *M*_*f*_, which acts on the posterior body. In the case of the swinging of the left leg, while the right leg is in the stance stage, the lizard must bring its left leg into the appropriate position by moving it by an amount equal to the distance from the position of the left leg at the first swing, which is called the stride length.

In order to implement the stride length for the left leg, the posterior body is required to rotate clockwise. Hence, a moment *M*_*swing*_, which acts on the posterior body, is required to realize the second swing. The posterior body is unable to rotate by itself, which implies that *M*_*swing*_ is required to be produced relative to the posterior body. Because *M*_*swing*_ is not equal to *M*_*f*_, the GRM (*M*_*z*_) generated by the twisting of the foot relative to the ground and the body torque (*T*_*bw*_, *T*_*bt*_) generated by the joint movement of the bodies relative to the posterior body are used to generate *M*_*swing*_. A lizard is able to run forward by repeating these steps.

### C. Dynamics analysis for the lizard’s undulatory body movement with optimization method

**Fig 3:**
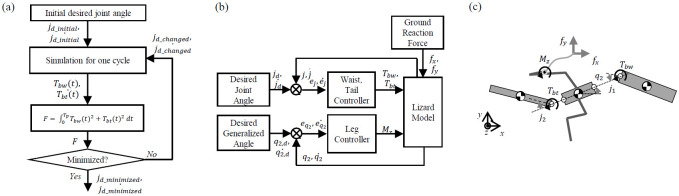
(a) Overall architecture of the dynamics analysis for benefits of the undulatory body movement, (b) simulation for one cycle of the gait of the lizard model with angular body movement (waist, tail, and leg controller are a proportional-derivative (PD) controller) and (c) forces and moments in the lizard model.

The dynamics analysis of the lizard theoretical model is performed according to the process in Fig 3(a). We set the initial desired value of the lizard body theoretical model. In the theoretical simulation, torque is generated in a proportional-derivative (PD) controller to follow the body joint’s initial desired angle. We extracted the lizard joint torque function over one cycle in the simulation. Next, we have defined a function that integrates the square of this torque value over one period. As a result, *j*_*d_minimized*_ of the body joint is derived when the function is minimized by changing the desired value of the lizard body joint through iteration.

After both the desired waist and tail movements are generated, the theoretical simulation can be formulated as shown in Fig 3(b). This type of periodic angular motion is assumed to be a sine function in their study on the design of a machine with fish-like biomimetic locomotion in a liquid environment [16]. The waist and tail movements of a lizard can be realized via the use of a sine function similar to the previous research method. These assumptions are not perfectly suited to mimic target animals but have the advantage of being able to reveal the effects and relationships of animal movements through simple calculations. The absolute angle of the posterior body is controlled by the GRM, but *T*_*bw*_ and *T*_*bt*_ generated by the waist and tail movements affect the magnitude of the GRM and the model’s running dynamics. GRM (*M*_*z*_), *T*_*bw*_, and *T*_*bt*_ are described in Fig 3(c).

According to Kubo *et al*. in [17], the pelvic rotation angle varies with the species of lizard and ranges from 11.7° to 25.8°. Reilly *et al*. observed the pelvic rotation to comprise a periodic sinusoidal movement [18]. The objective of our study is not to obtain a numerically perfect simulation of a running lizard, but to verify the effect of its lateral body undulation. Accordingly, the pelvic rotation is approximated as a sinusoidal movement of 20 sin(2*πt*/*T*_*p*_) ° (*T*_*p*_ = 0.092 s).

**Fig 4:**
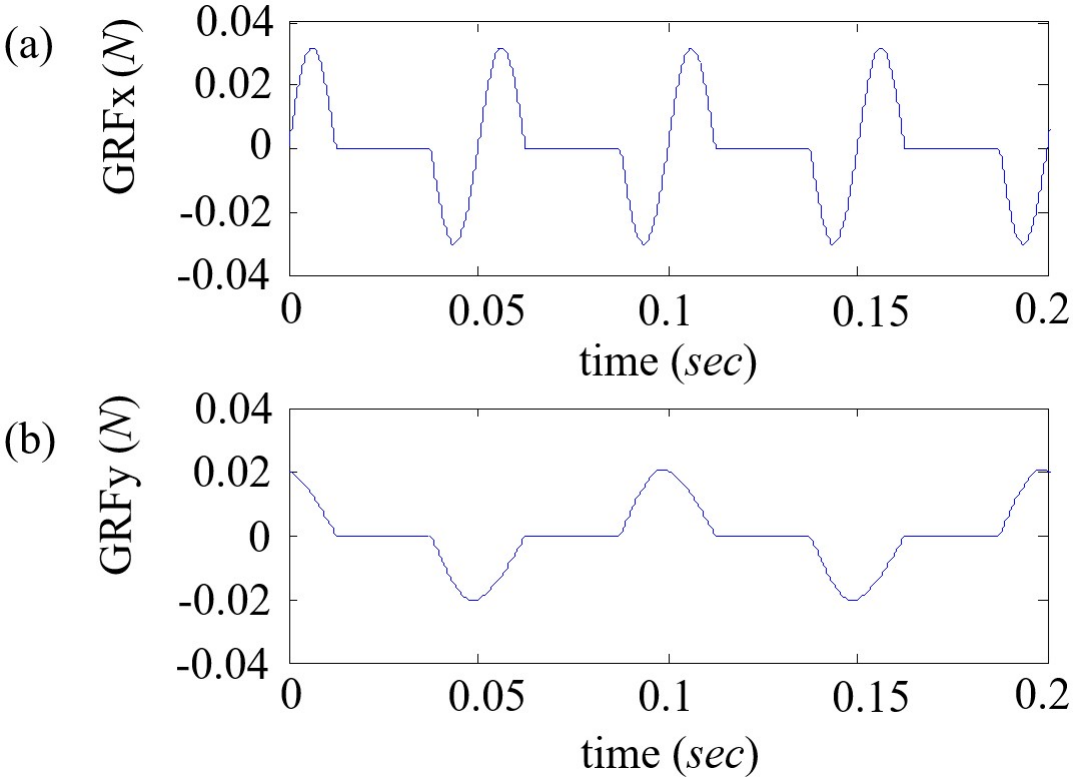
Profiles of (a) GRFx and (b) GRFy approximated as a sine function similar to the previous studies [20,21].

The magnitude of the GRF is mathematically derived while referring to related studies. GRFx, which acts in the forward-aft direction, was modeled as a sine function by Aerts *et al*. in [19]. The same mathematical derivation of GRFx is used in the simulation presented herein. In the case of GRFy, the direction of the force is always towards the midline of the body. The maximum value of GRFy is close to that of GRFx, whereas the impulse is much larger [20]. For the simulation, the direction, relative magnitude, and impact of GRFy are modeled according to the studies in [20,21], and mathematically formulated as a sine function. The GRF depends on the running speed of the lizard model, and thus, in the steady-state running, the GRF is periodically repeated in every stride. Therefore, the GRF was approximated as a sine function similar to the previous studies, as shown in Fig 4.

The position of the foot relative to the posterior body for every stride is determined from the leg length and angle data as presented in [13]. The location of the right foot is initially set as 38 mm away and at −45° from the center of mass of the posterior body, while the left foot is 38 mm away and at +45°. Without the loss of generality, the feet are modeled in a no-slip state during contact with the ground. The foot position is fixed, and the center of mass moves relative to the feet according to the GRF.

The movement of the waist and tail is minimized by the optimization, as shown in Fig 3(a). The desired angular movements of the waist and tail, which are assumed to be represented by sine functions, can be formulated as shown in Eq (1).

$$J_{d,i=1,2}=a_{i}\times \text{sin}(\frac{2\pi }{T_{P}}t+b_{i})$$ (1)

The angular body movement is achieved by combining the amplitude (*a*_*i*_) and phase difference (*b*_*i*_). As this study focuses on the effect of the body motion at high velocities, the frequency during running is fixed The angular movements of the waist and tail vary depending on the constants *a*_*i*_ and *b*_*i*_ in Eq (1). The values of the *a*_*i*_ and *b*_*i*_ determine the sinusoidal profile of the desired angular movement over time; then, the PD controller generates the waist and tail joints torques *T*_*bw*_ and *T*_*bt*_ to follow the desired profiles, as shown in Fig 3(b). Therefore, we determined *a*_*i*_ and *b*_*i*_ as the design parameters for the optimization. Also, we derived the objective function with the appropriate assumptions, as shown in Eq (2).

$$\text{Minimize}\;F(T_{bw}(t),T_{bt}(t))=\int_{0}^{T_{p}}[T_{bw}^{2}(t)+T_{bt}^{2}(t)]dt$$ (2)

Subject to GRM ≤ Const1 and $q_{2}=20\times \text{sin}(\frac{2\pi }{T_{p}}t)$.

The objective function to be minimized is set as (*T*_*bw*_^2^ + *T*_*bt*_^2^). The term (*T*_*bw*_^2^ + *T*_*bt*_^2^) can be expanded as ((*T*_*bw*_ + *T*_*bt*_)^2^ + (*T*_*bw*_ − *T*_*bt*_)^2^)/2, and thus, minimizing $\left(T_{bw}^{2}+{T_{bt}}^{2}\right)$ implies that the magnitude (*T*_*bw*_ + *T*_*bt*_) is minimized along with the uniform distribution of the moments to the joints via the term (*T*_*bw*_ − *T*_*bt*_). The moment generated at either the waist or tail joint acts as a burden on the active joint because it has to generate a relatively large moment. The uniform distribution of the moments over the joints reduces the load, and because a real lizard is presumed to act in the same manner, (*T*_*bw*_^2^ + *T*_*bt*_^2^) is selected as the objective function.

GRM (*M*_*z*_) is a constant that can be varied to produce different outcomes. Previously, we assume that the GRM (*M*_*z*_) produced by the lizard is very small. Thus, in the optimization process, the GRM (*M*_*z*_) is set to a minimum value. Here, *q*_2_ is the required absolute angle for the posterior body when the lizard is running at 4 m/s, as determined via a simple calculation comprising the stride length and time. We performed the optimization by sequential quadratic programming (SQP) using *fmincon* in MATLAB^®^.

### D. Result of the dynamic analysis for a lateral undulation of a bipedal running lizard

**Fig 5:**
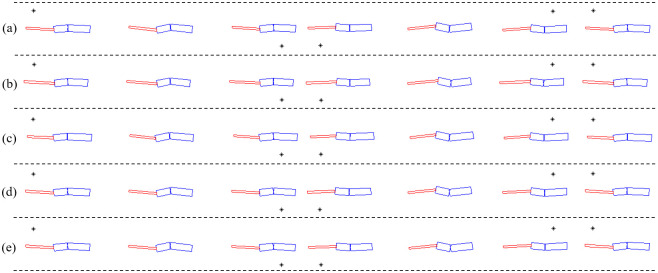
Movements of the lizard models calculated by the dynamics analysis when the joint torques are minimized. (* indicates the point at which the foot contacts the ground). (a) *l*_*1*_ = *66 mm*, *l*_*2*_ = *42 mm*, *l*_*3*_ = *84 mm*, *m*_*1*_ = *4*.*0 g*, *m*_*2*_ = *4*.*0 g*, *m*_*3*_ = *2*.*0 g*, (b) *l*_*1*_ = *60 mm*, *l*_*2*_ = *40 mm*, *l*_*3*_ = *92 mm*, *m*_*1*_ = *4*.*0 g*, *m*_*2*_ = *4*.*0 g*, *m*_*3*_ = *2*.*0 g*, (c) *l*_*1*_ = *72 mm*, *l*_*2*_ = *42 mm*, *l*_*3*_ = *78 mm*, *m*_*1*_ = *4*.*0 g*, *m*_*2*_ = *4*.*0 g*, *m*_*3*_ = *2*.*0 g*, (d) *l*_*1*_ = *66 mm*, *l*_*2*_ = *42 mm*, *l*_*3*_ = *84 mm*, *m*_*1*_ = *4*.*2 g*, *m*_*2*_ = *4*.*0 g*, *m*_*3*_ = *1*.*8 g*, (e) *l*_*1*_ = *66 mm*, *l*_*2*_ = *42 mm*, *l*_*3*_ = *84 mm*, *m*_*1*_ = *3*.*8 g*, *m*_*2*_ = *4*.*0 g*, *m*_*3*_ = *2*.*2 g*.

For various lizard theoretical models of different lengths and mass ratios, their lateral body undulations are generated in the simulations. We chose the size and weight of the lizard model based on the real lizard. It is worthwhile to note that the optimized motions may be affected by the size and weight of lizard model. Therefore, in this study, lizard models of different sizes and weights were used for simulations. The dynamic theoretical simulation is implemented in MATLAB^®^ with the numerical integration by the fourth-order Runge–Kutta method. The initial velocity of the model is set as *V*_*0*_ = 4 m/s. The model movement resulting from the optimization is presented in Fig 5. As expected, to minimize $\left(T_{bw}^{2}+{T_{bt}}^{2}\right)$, the waist and tail joints are required to generate moments that oppose each other, which results in a natural S-shaped movement of the body.

**Fig 6:**
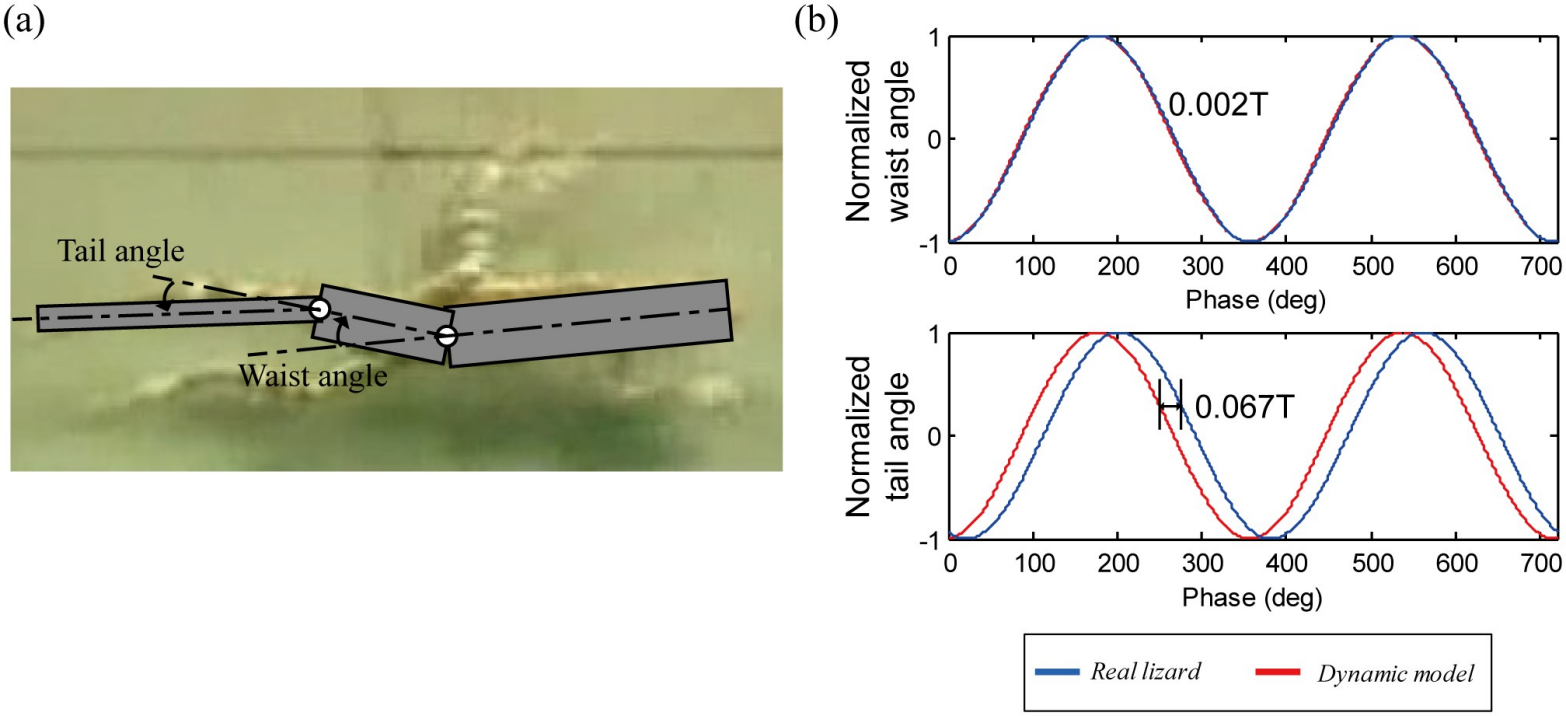
(a) Captured image of the running lizard for the joint angles of waist and tail from [22] and (b) comparison of the phase differences of the waist and tail joint angles between the real lizard and the dynamic model.

We have obtained the joint angles of the actual lizard’s waist and tail by capturing the images from a video file related with its running motion, as shown in Fig 6(a) from [22]. Then, we normalized them for comparison with the simulation. Since the shape of the lizard’s undulatory body movement is determined by the phases of the waist and tail movement angles, we compared the phase difference between the actual lizard’s and the simulated waist and tail joint angles as shown in Fig 6(b). The phase difference of the waist was 0.002T (T is the period), and the phase difference of the tail was 0.067T, indicating that the dynamics theoretical model and the real lizard’s waist and tail movements were very similar. In conclusion, we found that real lizards can minimize the sum of squares of torque by moving their body in an S-shape.

## 3. Lizard biomechanical modeling: For relationship between the undulatory body movement and the locomotion

In the previous section, we observe that the body movement of the lizard’s theoretical model when the joint torques are minimized. In addition, we have studied the relationship between the body movement and the joint torques of the theoretical model. In this section, to confirm the relationship between the lizard’s undulatory body movement and bipedal running locomotion, we established the lizard biomechanical model including its legs. To clearly identify the effect of the body movement, we have constructed a biomechanical simulation to generate the motion of the biomechanical model without control and to extract the joint torque through inverse dynamics, to exclude the effect of control performance.

### A. Bipedal running lizard biomechanical model

**Fig 7:**
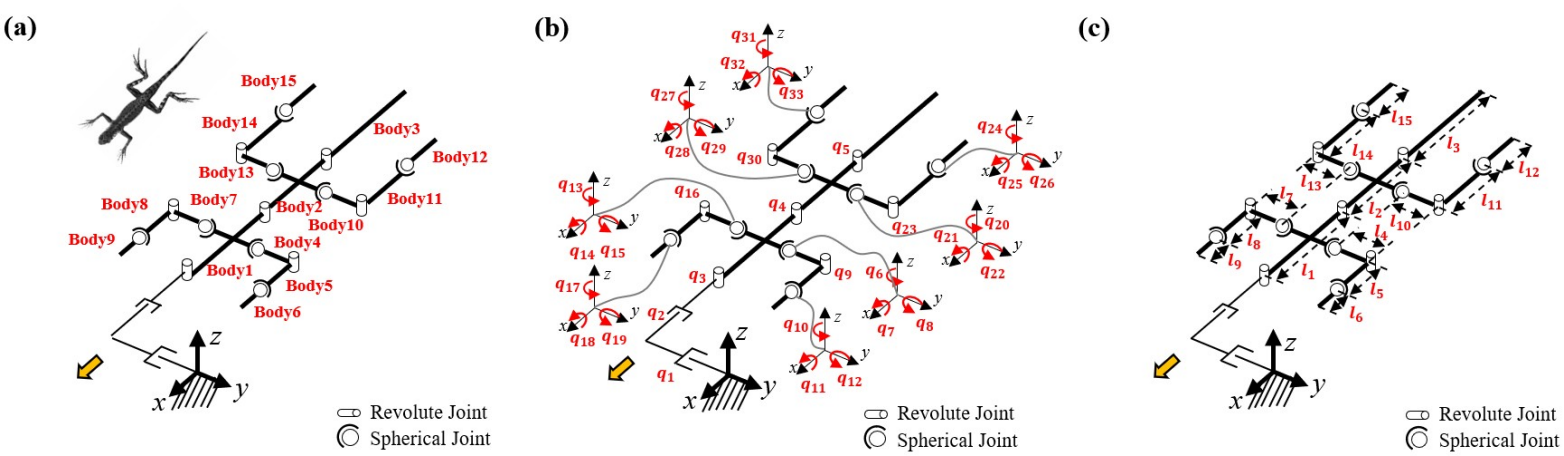
Kinematic biomechanical model of the lizard to confirm the relationship between the undulatory body movement and bipedal running locomotion: (a) bodies, (b) joints, and (c) lengths. The number of links used in the model is fifteen, and the number of joints is 33.

The biomechanical model of a bipedal running lizard is established by morphometrics of the *Callisaurus draconoides* based on [13], as in the previous section. The kinematics model is presented in Fig 7. The body of the model consists of three links referred to as body1, body2, and body3. In addition, the fore and hind legs of the biomechanical model comprise three links. The total number of the links used in the kinematics model of the lizard is fifteen, as shown in Fig 7(a).

The joints used for describing the angles of each body are set as rotational joints with one DOF. The fore and hind legs comprise shoulder, knee, and ankle joints. The shoulder and ankle joints are spherical joints with three DOFs. The knee joint is a rotational joint with one DOF. We add two linear joints as virtual joints to calculate the position of the biomechanical model in the fixed coordinate frame. The number of joints in the kinematics model is 33, as shown in Fig 7(b). The length and mass of the links of the biomechanical model, based on [13], are listed in Table 2.

**Table 2:** Size and mass of a lizard biomechanical model.

Lizard model links	Length (mm)	Width (mm)	Mass (g)
Body1	66	16.5	3.6
Body2	42	13	2.9
Body3	84	4.5	2.0
Body4, 7	16	4	0.11
Body5, 8	12	3	0.06
Body6, 9	15	3	0.03
Body10, 13	19	7.5	0.36
Body11, 14	21	4.5	0.18
Body12, 15	13	3.5	0.08

The center of the mass of each link is in the middle of the link. The positions of the center of the mass are explained in the Appendix A2 using the lengths and the joint angles of the lizard biomechanical model.

### B. Joint angles of the biomechanical model for the bipedal running locomotion

We simulate the locomotion and joint movement of the lizard’s biomechanical model. In this simulation, the joint angles of the robot model are simplified as a sine function according to the actual lizard’s joint angle. The sine functions of the joint motion are defined as follows:

$$q_{i}=a_{i}\times \text{sin}(\frac{2\pi }{\text{period}}t+b_{i})+c_{i},\;i=3,\cdots ,33$$ (3)

where *a*_*i*_ is the magnitude, *b*_*i*_ is the phase difference, and *c*_*i*_ is the offset of the sine function. As we change these variables, the joint angle of the model changes dramatically.

The movement of the biomechanical model is laterally symmetric because it is assumed that the model runs in a straight line without any deviation. To realize symmetric movement in the lateral direction, the body joint angle has two variables of magnitude *a*_*i*_ and phase difference *b*_*i*_ without an offset *c*_*i*_. In addition, the left and right hind legs perform the same symmetric movement with a 180° phase difference. The joints of the forelegs are fixed at a constant angle to mimic a real lizard’s posture. We summarized the motion of each joint in Table 3.

**Table 3:** Joint motion of the lizard biomechanical model.

Joint’s name	Symbol	Joint motion
Waist, tail joint	*q*_4_, *q*_5_	$q_{i}=a_{i}\times \text{sin}(\frac{2\pi }{\text{period}}t+b_{i})$ (4)
Shoulder X, Z axis & Knee of Left hind leg	*q*_20_, *q*_21_ *q*_22_	$q_{i}=a_{i}\times \text{sin}(\frac{2\pi }{\text{period}}t+b_{i})+c_{i}$ (5)
Shoulder X, Z axis & Knee of Right hind leg	*q*_27_, *q*_28_ *q*_29_	$q_{i}=a_{i-7}\times \text{sin}(\frac{2\pi }{\text{period}}t+b_{i-7})-c_{i-7}$ (6)
Shoulder Y axis of Left hind leg	*q*_22_	$q_{i}=a_{i}\times \text{sin}(\frac{2\pi }{\text{period}}t+b_{i})+c_{i}$ (7)
Shoulder Y axis of Right hind leg	*q*_29_	$q_{i}=a_{i-7}\times \text{sin}(\frac{2\pi }{\text{period}}t+b_{i-7}+\pi )-c_{i-7}$ (8)
Fore leg’s joints	*q*_*i*_, *i* = 6,⋯,19	*q*_*i*_ = *c*_*i*_ (9)

The ankle joint of the lizard model is set with the foot always facing forward. This setting is determined by observing that the foot of a real lizard faces forward when the lizard’s foot touches on the ground. Therefore, we set the ankle joint angle to x, z = 0 and fix the y-axis value in the fixed coordinate frame, such that lizard’s foot is facing forward when it touches the ground at any time.

In the biomechanical simulation, it is assumed that the body of the model is maintained at a constant distance from the ground. The lizard’s body is observed to have a small up and down movement [12,15,23]. Thus, in order to simplify the simulation and focus on the lateral body undulation of the biomechanical model, we assume that the body of the biomechanical model only moves in the horizontal plane.

The distance between the body of the biomechanical model and the ground is determined based on the trajectory of the hind legs. Based on the body position, the z-direction position of the ground should be between the minimum of the foot end and the minimum of the hind ankle. If the ground position is lower than the minimum position of the foot end of the hind foot, the hind foot of the model cannot reach the ground. On the other hand, if the ground position is higher than the minimum position of the hind ankle, then the model’s ankle touches the ground, which is different from the actual lizard’s movement. Therefore, in this study, we set the ground position of the body from the center of the foot at the minimum foot position in the z-direction.

**Fig 8:**
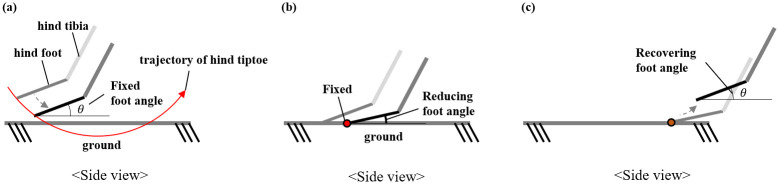
Changes in the angle of the ankle joint while the hind legs touch the ground. (a) before the foot touches the ground, (b) while the foot touches the ground, and (c) after the foot falls off the ground.

Before the hind foot touches the ground, the posture of the foot is fixed, as described in the previous section and as shown in Fig 8(a). When the position of the foot end of the hind foot is lower than the position of the ground, the angle of the ankle is changed such that the position of the foot end is the same as the position of the ground, as shown in Fig 8(b). If the position of the foot end of the back foot is higher than the position of the ground, the hind foot returns to its original posture as shown in Fig 8(c). Therefore, when the foot touches the ground, the position of the foot is fixed, and after the foot leaves the ground, it returns to its original posture. While in contact with the ground, the movement of the model is implemented based on the fixed position of the foot end of the hind leg.

When the model is in the air without contacting the ground, we assume that the center of the mass of the biomechanical model moves linearly. Since the biomechanical model is not forced in the air, the velocity of the model does not change. The velocity and the direction of the model in the air are calculated from the previous velocity and direction before falling off the ground.

### C. Inverse dynamics of the lizard biomechanical model

**Fig 9:**
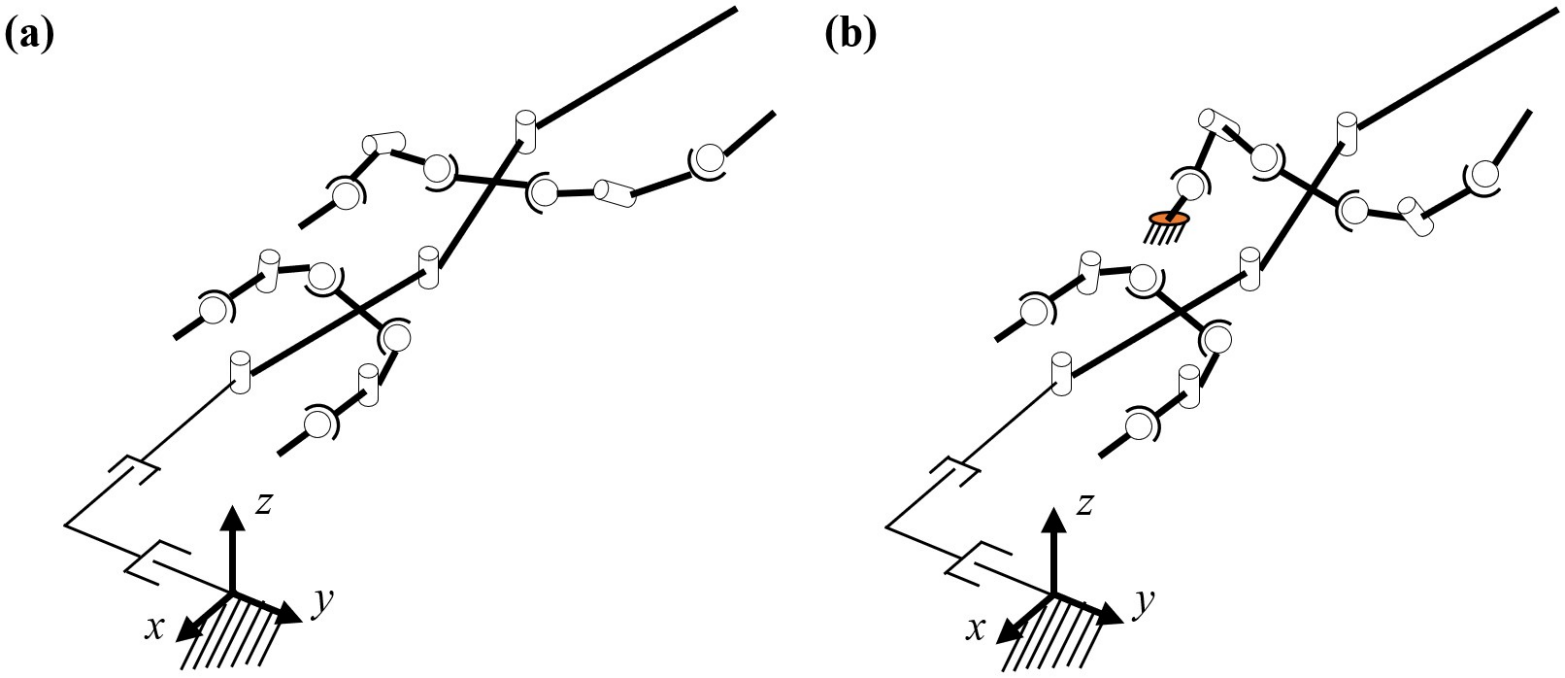
The dynamic system of the lizard biomechanical model: (a) Non-contact, (b) contact with the ground. The position of the tiptoe is fixed while the foot touches on the ground.

The number of joints in the biomechanical model is k = 30 and with three virtual joints, the total number of joints is n = k+3 = 33. When the biomechanical model does not touch the ground as shown in Fig 9(a), the equation of motion is derived as follows.

$$A(q)\ddot{q}+b(q,\dot{q})+g(q)=\Gamma$$ (10)

where *q* ∈ *R*^33×1^ is the joint vector and Γ ∈ *R*^33×1^ is the torque vector. *A*(*q*) ∈ *R*^33×33^ is the mass and inertia matrix and $b(q,\dot{q})\in R^{33\times 1}$ is the Coriolis and centrifugal vector.*g*(*q*) ∈ *R*^33×1^ is the gravity vector. When the foot of the lizard biomechanical model contacts the ground, as shown in Fig 9(b), the reaction forces and moments are taken into consideration in the equation of motion from the ground as follows.

$$A(q)\ddot{q}+b(q,\dot{q})+g(q)+J_{c}^{T}f_{c}=\Gamma$$ (11)

$J_{c}^{T}$ is the transpose of the Jacobian of the position and the angle at the foot on the ground. In addition, *f*_*c*_ is the reaction force and moment vector.*J*_*c*_ must satisfy Eq (12).

$${\dot{x}}_{c}=J_{c}\dot{q}$$ (12)

where *x*_*c*_ is the position vector of the foot that contacts the ground and *q* is the joint vector. Then, by replacing $\ddot{q}$ with $\ddot{q}=J_{c}^{-1}(\ddot{x_{c}}-\dot{J_{c}}\dot{q})$, Eq (11) becomes

$$\begin{array}{l} A(q)J_{c}^{-1}({\ddot{x}}_{c}-{\dot{J}}_{c}\dot{q})+b(q,\dot{q})+g(q)+J_{c}^{T}f_{c}=\Gamma \\ \Leftrightarrow \Lambda_{c}{\ddot{x}}_{c}+\Lambda_{c}(J_{c}A^{-1}(q)b(q,\dot{q})-{\dot{J}}_{c}\dot{q})+\Lambda_{c}J_{c}A^{-1}(q)g(q)+f_{c}=\Lambda_{c}J_{c}A^{-1}(q)\Gamma \end{array}$$ (13)

*f*_*c*_ can be derived from Eq (13) via translation from the joint space to the operational space at the foot position on the ground as follows:

$$\Lambda_{c}{\ddot{x}}_{c}+\mu_{c}+p_{c}+f_{c}={\bar{J}}_{c}^{T}\Gamma$$ (14)

where,

$$\Lambda_{c}={\left(J_{c}A^{-1}(q)J_{c}^{T}\right)}^{-1}$$ (15)

$$\mu_{c}=\Lambda_{c}\{J_{c}A^{-1}(q)b(q,\dot{q})-{\dot{J}}_{c}\dot{q}\}$$ (16)

$$p_{c}=\Lambda_{c}J_{c}A^{-1}(q)g(q)$$ (17)

$${\bar{J}}_{c}^{T}=\Lambda_{c}J_{c}A^{-1}(q)$$ (18)

Λ_*c*_ is the inertia and mass matrix in the operational space. $\overset{-}{J_{c}}$ is dynamically consistent with the inverse vector of *J*_*c*_. *μ*_*c*_ is the Coriolis and centrifugal vector in the operational space.*p*_*c*_ is the gravity vector in the operational space. When the foot is fixed on the ground, $\dot{x_{c}}$ and $\ddot{x_{c}}$ are zero. Therefore, the reaction force and the moment vector *f*_*c*_ is derived as follows:

$$f_{c}={\bar{J}}_{c}^{T}\Gamma -\mu_{c}-p_{c}$$ (19)

on substituting Eq (11) in Eq (19), the equation of motion is derived as follows:

$$A(q)\ddot{q}+b(q,\dot{q})+g(q)+J_{c}^{T}\{{\bar{J}}_{c}^{T}\Gamma -\mu_{c}-p_{c}\}=\Gamma$$ (20)

The angular velocity and acceleration are obtained from the differential of the joint angle and angular velocity. We have calculated the torque using Eq (20) with the angular velocity, acceleration, and the masses of the links.

## 4. Simulation results

**Fig 10:**
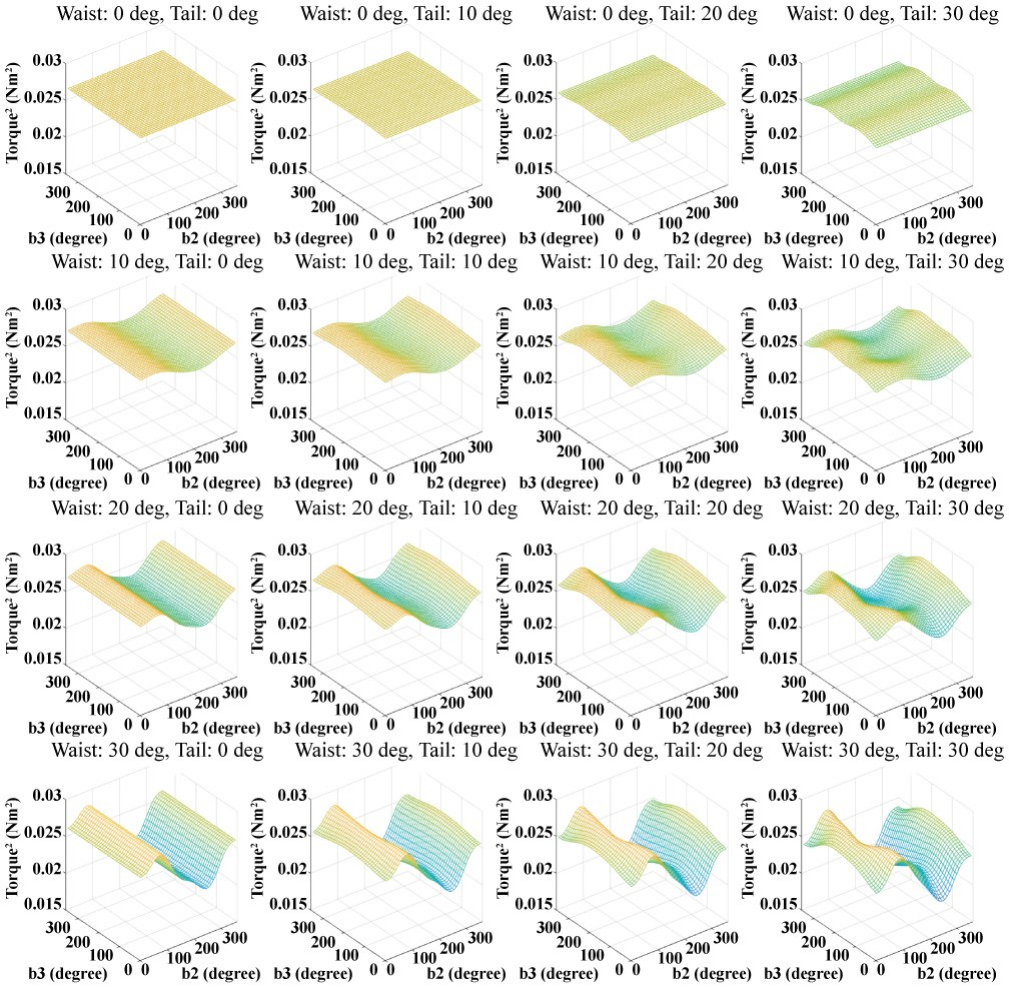
Squares of the torques of all joints obtained by varying the body movement. The magnitude of the waist joint motion changes in the vertical direction, and the magnitude of the tail joint motion changes in the horizontal direction.

The joint torque can be obtained from the inverse dynamics of the biomechanical model when the model runs. As the biomechanical model body’s motion changes, all joint torques change. Fig 10 shows the average of the squares of the joint torques obtained by changing the waist and tail joint angle. In Fig 10, the waist joint motion affects the square of the joint torque to a greater extent than the tail joint motion. As the amplitude of the waist joint motion is greater, the effect of the phase difference of the waist joint motion is also greater. The undulatory body movement of the biomechanical model has the significant effect on the square of the joint torque. This means that it is important to determine the appropriate undulatory body movement for minimizing the joint torques of the lizard.

In the previous section, it is observed that the lizard biomechanical model’s body moves similar to the actual lizard when the square of the joint torque is minimized. In this section, we select the square of the joint torque as the cost function of the optimization as follows:

$$\text{Minimize}\;F(\Gamma )=\int_{0}^{\text{period}}\sum \|\Gamma_{i}{}^{2}\|dt,i=4,5,\cdots ,33$$ (21)

As described in the previous section, the motion angles of the joint of the lizard biomechanical model are simplified as the sine functions. When some variables are fixed based on assumptions, the number of variables that change the body and leg movements is reduced to nine as shown in Table 4. Thus, we select these variables as the design parameters for minimizing the cost function.

**Table 4:** Design parameters for optimization.

Order	Description	Variable
1	Magnitude of the waist joint angle	*a*_*4*_
2	Phase difference of the waist joint angle	*b*_*4*_
3	Magnitude of the tail joint angle	*a*_*5*_
4	Phase difference of the tail joint angle	*b*_*5*_
5	Magnitude of the shoulder z joint angle	*a*_*20*_
6	Offset of the shoulder z joint angle	*b*_*20*_
7	Magnitude of the shoulder x joint angle	*a*_*21*_
8	Magnitude of the shoulder y joint angle	*a*_*22*_
9	Magnitude of the knee joint angle	*a*_*23*_

**Fig 11:**
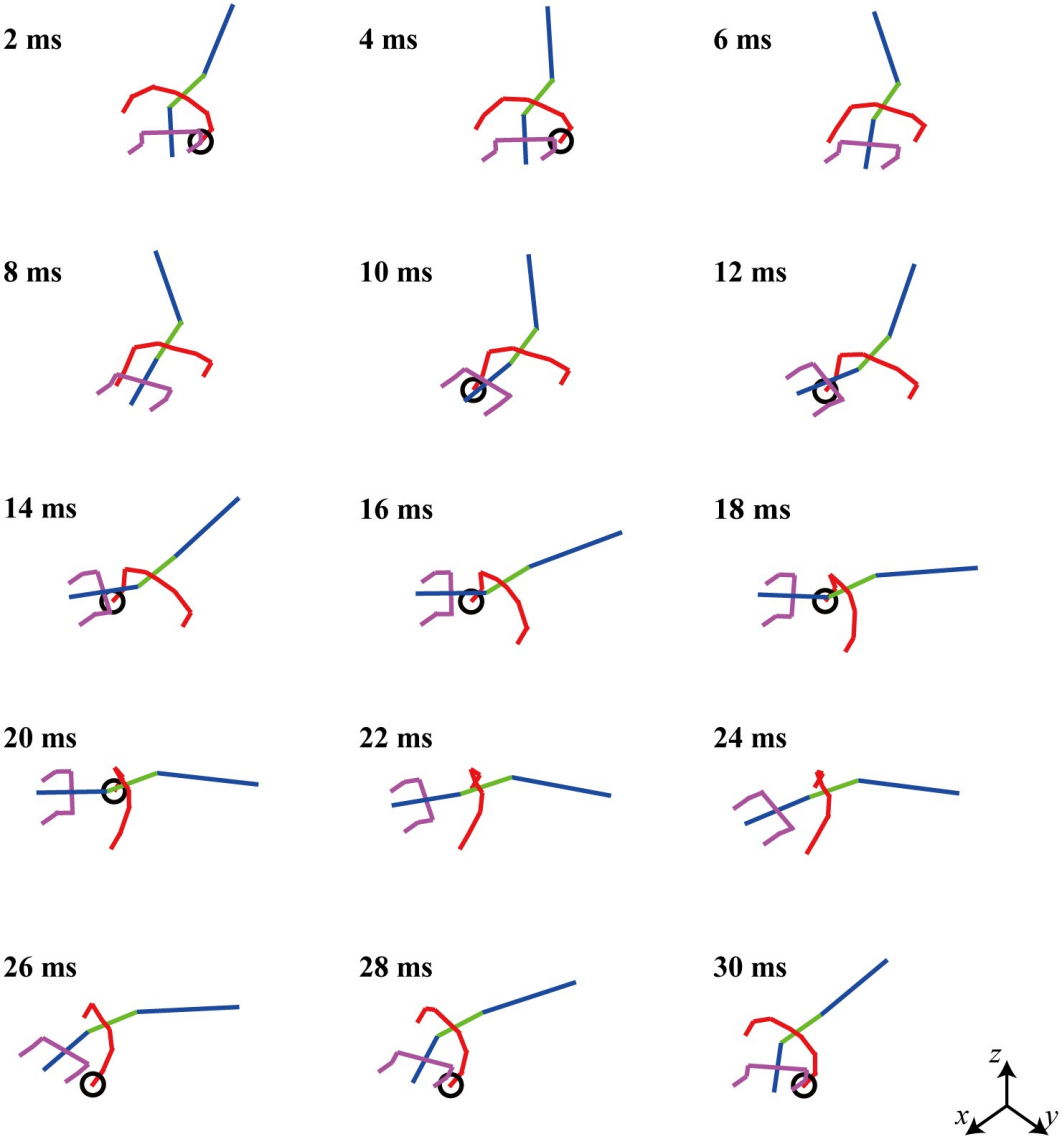
Snapshot of a moving video clip of a simulated lizard when the square of the joint torque is minimized. The lizard biomechanical model has bipedal running locomotion at a speed of 4 m/s.

Fig 11 presents snapshots of the locomotion video clip of the biomechanical simulation model derived via the optimization.

The pelvic angle of the biomechanical model is assumed to be 20 sin(2*πt*/*T*_*p*_), which is the same as that in the previous section II. The velocity of the simulation biomechanical model is 4 m/s, and the period time (= *T*_*p*_) is 0.03 s. The right and left legs repeat the same movement with a 180° phase difference. The phase difference of the waist joint angle is different from that of the tail joint angle. In Fig 11, it appears that the waist joint moves first, followed by the tail joint. For the readers, the MATLAB code used for the simulation in Fig 11 can be accessed with the instruction file (https://github.com/imationzzz/LizardSimulation).

**Fig 12:**
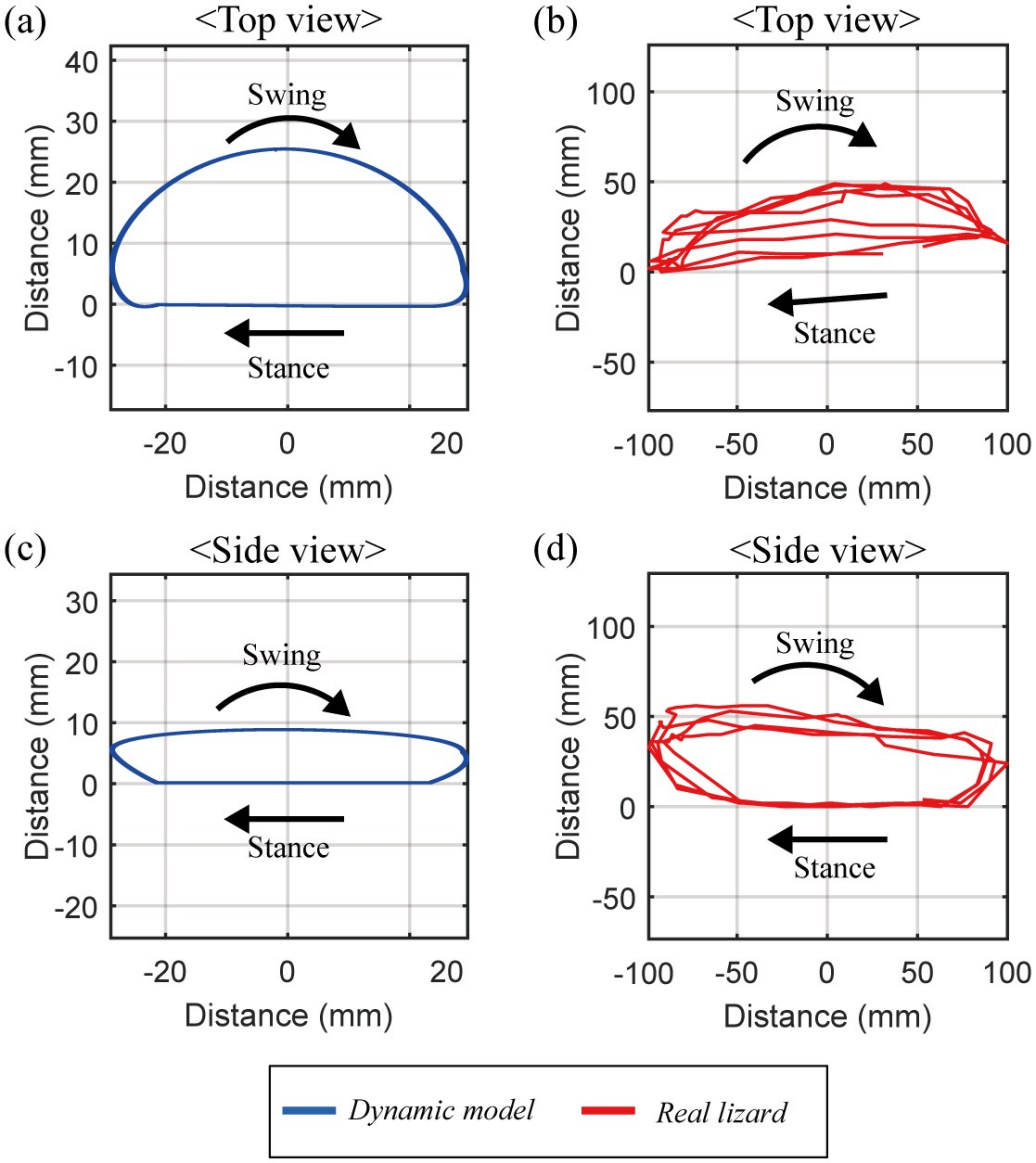
Comparison of the tiptoe’s trajectory between the dynamic biomechanical model and the real lizard. (a) The trajectory of the tiptoe of the lizard biomechanical model in the top view has a length of 58.5 mm in the horizontal direction, and 25.8 mm in the vertical direction. (b) The trajectory of the tiptoe of the real lizard in the top view has a length of 199 mm in the horizontal direction, and 49 mm in the vertical direction. (c) The trajectory of the tiptoe of the lizard biomechanical model in the side view has a length of 58.5 mm in the horizontal direction, and 8.7 mm in the vertical direction. (b) The trajectory of the tiptoe of the real lizard in the side view has a length of 199 mm in the horizontal direction, and 56 mm in the vertical direction.

Fig 12 shows the trajectory of the left hind tiptoe of the lizard biomechanical model running at 4 m/s in place and compares it with that of the actual lizard captured from its running locomotion [22]. As shown in Fig 12(a) and 12(b), in the stance mode, both the lizard biomechanical model and the real lizard move their tiptoes back in a straight line but bring them forward in the swing mode. The trajectory length of the tiptoe of the lizard biomechanical model in the top view is 58.5 mm in the horizontal direction, and its maximum width is 25.8 mm in the vertical direction. On the other hand, the trajectory length of the real lizard in the top view is 199 mm in the horizontal direction, and its maximum width is 49 mm in the vertical direction. As shown in Fig 12(c) and 12(d), both the lizard biomechanical model and the real lizard raise their tiptoes upward in swing mode. The trajectory length of the lizard biomechanical model in the side view is 58.5 mm in the horizontal direction, and its maximum height is 8.7 mm in the vertical direction. The trajectory length of the real lizard in the side view is 199 mm in the horizontal direction, and its maximum height is 56 mm in the vertical direction. Through this simulation, we found that when the lizard biomechanical model ran in the bipedal locomotion with minimizing the sum of squares of the torque, the resulting trajectory of tiptoe is similar to that of the actual lizard even though their sizes are different.

**Fig 13:**
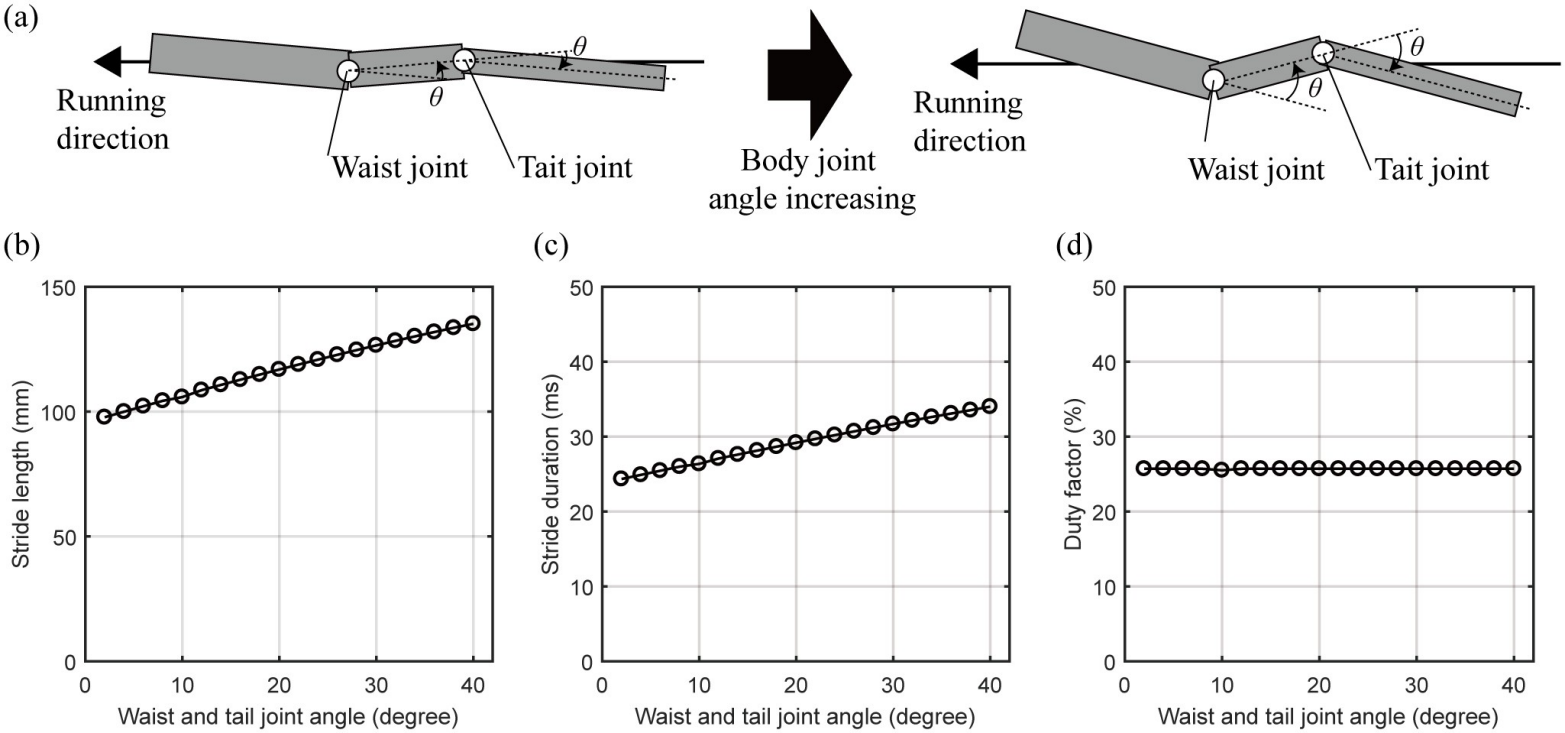
The locomotion characteristics of the lizard biomechanical model changed by increasing the undulatory body movement. (a) Concept of the increase of the lizard’s undulatory body movement. The waist and tail joint angles are set to the same angle. (b) The stride length and (c) duration are increased by increasing the lizard’s undulatory body movement. (c) The duty factor does not be changed by the undulatory body movement of the lizard biomechanical model.

We performed a simulation to confirm the bipedal running locomotion of the lizard biomechanical model by gradually increasing the size of the waist and tail joint angle, as shown in Fig 13(a). The body movement is a sine function, and the waist and tail joints are set to be same. Excluding the waist and tail joints, the legs’ movements were derived to minimize the square of the torque, as in the previous simulation. As a result, the characteristics of locomotion were obtained, as shown in Fig 13(b)–13(d). When the lizard biomechanical model runs at the same speed, Fig 13(b) shows that the stride length increases from 97.8 mm to 135.2 mm as the body movement increases from 2 degrees to 40 degrees. The stride duration also increases from 24.3 ms to 34.0 ms (see Fig 13(c)). The stride frequency is the reciprocal number of the stride duration and decreases from 41.2Hz to 29.4Hz. Therefore, as the body movement increases, the stride length increases, so the lizard can achieve a high running speed with fewer moves. On the other hand, Fig 13(d) shows that the duty factor of the lizard maintains its value, about 25.7%, independent of the body’s movement.

When the joint angle of the lizard’s waist and the tail are 40 degrees, the characteristics of simulated locomotion are compared with those of actual lizard obtained from [13] in Table 5. The stride length of the lizard biomechanical model is smaller than that of the real lizard. It is guessed that the difference between the simulation model and the real lizard may stem from the modeling error. On the other hand, since other values are similar between the biomechanical model and the lizard, the modeling error may be negligible.

**Table 5:** Comparison of locomotion specification between the biomechanical model and the real lizard.

Variable	Lizard model with 40° of body joint	*Callisaurus draconoides* [13]
Speed (m/s)	4.0	4.0±0.1
Stride length (mm)	135.2	319±11
Stride width (mm)	40.9	51±4
Stride duration (ms)	34.0	80±3
Duty factor (%)	25.7	24±1
Hip height (mm)	30.3	28±1

## 5. Conclusion

This study shows that the steady-state bipedal straight running of a lizard is highly related to the symmetric and periodical angular movement of its posterior body. It is hypothesized that such pelvic rotation is produced owing to the GRM and internal torque. The dynamic theoretical simulation is used to discover the relationship between the GRM and internal torque. In the lizard theoretical model of bipedal and steady-state running, the GRF associated with the running speed is assumed to be identical for every stride based on previous studies. When the GRM, torque distribution, and torque magnitude are minimized, an S-shaped lateral body undulation similar to that of a real lizard is observed in the model.

Furthermore, we established the lizard biomechanical model to figure out the relationship between the lizard’s undulatory body movement and the bipedal running locomotion. The biomechanical model consists of 15 bodies and 33 DOFs. When the biomechanical model performs running in bipedal locomotion, the square of the joint torque is changed by the waist and tail joints. It means that the lizard’s undulatory body movement significantly affects all joint torques when the lizard performs bipedal running locomotion. When the square of the joint torque was minimized, we observed that the undulatory body movement are quite similar to those of a lizard. The trajectory of the lizard’s biomechanical model tiptoe is small than a real lizard’s trajectory; however, the trajectory shape is similar to a real lizard’s tiptoe trajectory. We performed a simulation to confirm the bipedal running locomotion of the lizard biomechanical model by gradually increasing the size of the waist and tail joint angle. As a result, when the lizard biomechanical model runs at the same speed, the stride length and stride duration increase, and the stride frequency decrease. Therefore, as the body movement increases, the lizard can achieve a high running speed with fewer moves. It means that the lizard’s undulatory body movements increase its stride and help it run faster.

As a result, we identify the benefits of the lizard-body movements that minimize the square of the joint torque. Besides, we confirmed that the undulatory body movements of lizard affect the lizard’s locomotion. To the best of our knowledge, there is no research to figure out the principle of the running lizard’s undulatory body movement and relationship with the locomotion. In future work, we will analyze body movements in various ways through various environments and simulators.

## 6. Appendix: A1

**Table:** Nomenclature.

Symbol	Description
*x*_*i* = 1, 2, 3_	*x* position of COM of links
*y*_*i* = 1, 2, 3_	*y* position of COM of links
*m*_*i* = 1, 2, 3_	mass of links
*l*_*i* = 1, 2, 3_	length of links
*w*_*i* = 1, 2, 3_	width of links
*q*_*i* = 1, 2, 3_	generalized angle of link *i*
*j*_*i* = 1, 2_	angle of joint *i*
*u*_*i* = 1, 2_	actuator torque of joint *i*
*f*_*i* = 1, 2_	joint constraint force of joint *i*
*M*_*z*_	reaction moment in z direction from ground
*p*_*x*_	x component of the distance between the COM of the posterior body and foot
*p*_*y*_	y component of the distance between the COM of the posterior body and foot

**Fig 14:**
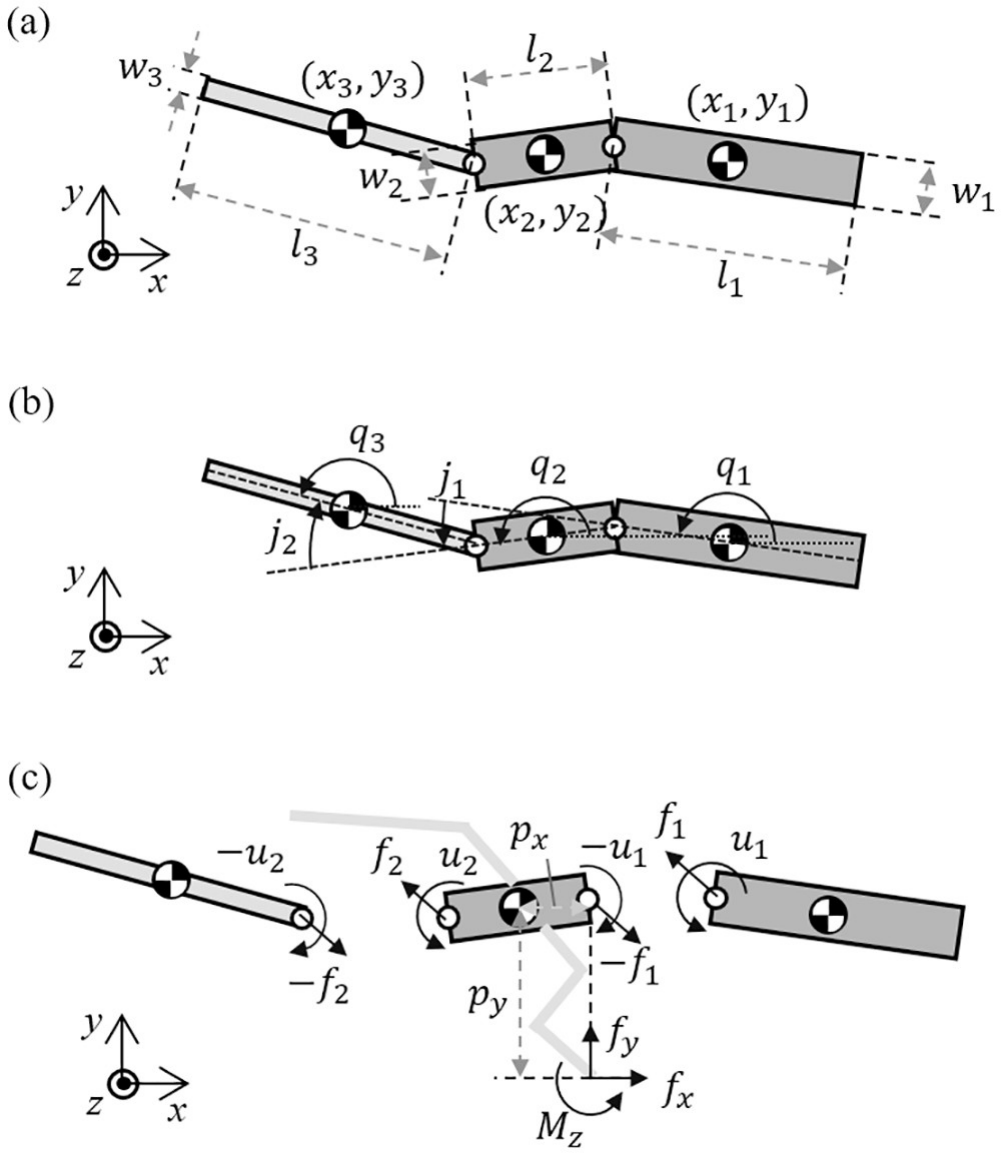
(a) Kinematic parameters for the lizard theoretical model, (b) the generalized angle of the links and the relative angle between the links and (c) forces and moments acting on each link of the lizard theoretical model in the horizontal plane.

As shown in Fig 14, for dynamic equation, the vectors for position, angle, mass and moment of inertia are defined in *R*^3×1^. The horizontal position vector of center of mass of the link is defined as *x* = [*x*_1_, *x*_2_, *x*_3_]^*T*^ and the lateral position vector is defined as *y* = [*y*_1_, *y*_2_, *y*_3_]^*T*^. The angle vector of the link in global frame is *q* = [*q*_1_, *q*_2_, *q*_3_]^*T*^, the joint angle vector between each links is *j* = [*j*_1_, *j*_2_]^*T*^. The mass vector is defined as *m* = [*m*_1_, *m*_2_, *m*_3_]^*T*^. For numerical calculation, *M* = *diag*(*m*) is defined. The moment of the inertia vector is defined by *i* = [*i*_1_, *i*_2_, *i*_3_]^*T*^ and in the same manner, *I* = *diag*(*i*). The sine and cosine vectors are defined as sin*q* = [sin*q*_1_, sin*q*_2_, sin*q*_3_]^*T*^, Sin*q* = *diag*(sin*q*), cos*q* = [cos*q*_1_, cos*q*_2_, cos*q*_3_]^*T*^ cos*q* = *diag*(cos*q*).

The model has a boundary as shown in Fig 14 ([24,25])

$$x_{i+1}-x_{i}=\frac{1}{2}(l_{i+1}\text{cos}q_{i+1}+l_{i}\text{cos}q_{i})$$ (22)

$$y_{i+1}-y_{i}=\frac{1}{2}(l_{i+1}\text{sin}q_{i+1}+l_{i}\text{sin}q_{i})$$ (23)

The following are the vector forms of Eqs (22) and (23)

$$Cx=\frac{L}{2}\text{cos}q$$ (24)

$$Cy=\frac{L}{2}\text{sin}q$$ (25)

*C* and *L* are defined in Eqs (26) and (27).

$$C=[\begin{array}{cccc} -1 & 1 & 0 & 0\\ 0 & -1 & 1 & 0\\ 0 & 0 & -1 & 1 \end{array}]$$ (26)

$$L=[\begin{array}{cccc} l_{1} & l_{2} & 0 & 0\\ 0 & l_{2} & l_{3} & 0\\ 0 & 0 & l_{3} & l_{4} \end{array}]$$ (27)

The equation of motion of the theoretical model is derived as

$$m_{i}{\ddot{x}}_{i}=f_{ext,x,i}+f_{x,i}-f_{x,i-1}$$ (28)

$$m_{i}{\ddot{y}}_{i}=f_{ext,y,i}+f_{y,i}-f_{y,i-1}$$ (29)

The vector forms of Eqs (28) and (29) are as follow:

$$M\ddot{x}=F_{\text{ext},x}+C^{T}F_{x}$$ (30)

$$M\ddot{y}=F_{\text{ett},y}+C^{T}F_{y}$$ (31)

From the assumption that the reaction force exists only in hind leg, the vectors of the reaction force are *F*_*ext*,*x*_ = [0, *f*_*ext*,*x*_, 0]^*T*^, *F*_*ext*,*v*_ = [0, *f*_*ext*,*v*_, 0]^*T*^. The inner force vector are defined as *F*_*x*_ = [*f*_*x*1_, *f*_*x*2_]^*T*^ and *F*_*v*_ = [*f*_*v*1_, *f*_*v*2_]^*T*^.

Eqs (32) and (33) are derived by differentiating Eqs (24) and (25) twice:

$$C\ddot{x}=-\frac{L}{2}(\text{Cos}q\cdot {\dot{q}}^{2}+\text{Sin}q\cdot \ddot{q})$$ (32)

$$C\ddot{y}=\frac{L}{2}(-\text{Sin}q\cdot {\dot{q}}^{2}+\text{Cos}q\cdot \ddot{q})$$ (33)

From Eqs (30)–(33), the inner force vectors are derived as

$$F_{x}={\left(CM^{-1}C^{T}\right)}^{-1}\{-\frac{L}{2}(\text{Cos}q\cdot {\dot{q}}^{2}+\text{Sin}q\cdot \ddot{q})-CM^{-1}F_{ext,x}\}$$ (34)

$$F_{y}={\left(CM^{-1}C^{T}\right)}^{-1}\{\frac{L}{2}(\text{Sin}q\cdot {\dot{q}}^{2}+\text{Cos}q\cdot \ddot{q})-CM^{-1}F_{ext,y}\}$$ (35)

The equation of moment of the theoretical model is derived as

$$I\ddot{q}=C^{T}u-\frac{L}{2}\text{Sin}q\cdot F_{x}+\frac{L}{2}\text{Cos}q\cdot F_{y}+D_{y}F_{xt,x}+D_{x}F_{et,y}+M_{ext,z}$$ (36)

where *u* = [*u*_1_, *u*_2_]^*T*^ is the joint torque vector. *D*_*x*_ = [0, *p*_*x*_, 0]^*T*^ and *D*_*v*_ = [0, *p*_*v*_, 0]^*T*^ are the vectors of the distance from the center of body2 to the tip of the hind leg. *M*_*ext*.*z*_ = [0, *M*_*z*_, 0]^*T*^ is defined as the vector of the reaction moment on the ground.

## 7. Appendix: A2

*X*_*i*_ is a position of the center of the mass of *body*_*i*_ in the biomechanical model.

$$X_{1}=[\begin{array}{c} q_{1}\\ q_{2}\\ 0 \end{array}]+Rot(q_{3},z)\times [\begin{array}{l} \frac{l_{1}}{2}\\ 0\\ 0 \end{array}]$$ (37)

$$X_{2}=X_{1}+Rot(q_{3},z)\times (\left[\begin{array}{c} \frac{l_{1}}{2}\\ 0\\ 0 \end{array}]+Rot(q_{4},z)\times [\begin{array}{c} \frac{l_{2}}{2}\\ 0\\ 0 \end{array}\right])$$ (38)

$$X_{3}=X_{2}+Rot(q_{3},z)\times Rot(q_{4},z)\times (\left[\begin{array}{c} \frac{l_{2}}{2}\\ 0\\ 0 \end{array}]+Rot(q_{5},z)\times [\begin{array}{l} \frac{l_{3}}{2}\\ 0\\ 0 \end{array}\right])$$ (39)

$$X_{4}=[\begin{array}{c} q_{1}\\ q_{2}\\ 0 \end{array}]+Rot(q_{3},z)\times [\begin{array}{c} \frac{l_{2}}{2}+shoulderX\\ -shoulderY\\ 0 \end{array}]+Rot(q_{3},z)\times Rot(q_{6},z)\times Rot(q_{7},x)\times Rot(q_{8},y)\times [\begin{array}{c} 0\\ \frac{-l_{4}}{2}\\ 0 \end{array}]$$ (40)

$$X_{5}=X_{4}+Rot(q_{3},z)\times Rot(q_{6},z)\times Rot(q_{7},x)\times Rot(q_{8},y)\times (\left[\begin{array}{c} 0\\ \frac{-l_{4}}{2}\\ 0 \end{array}]+Rot(q_{9},z)\times [\begin{array}{c} \frac{-l_{5}}{2}\\ 0\\ 0 \end{array}\right])$$ (41)

$$\begin{array}{ll} X_{6} & =X_{5}+Rot(q_{3},z)\times Rot(q_{6},z)\times Rot(q_{7},x)\times Rot(q_{8},y)\times Rot(q_{9},z)\times [\begin{array}{c} \frac{-l_{5}}{2}\\ 0\\ 0 \end{array}]\\ & +Rot(q_{10},z)\times Rot(q_{11},x)\times Rot(q_{12},y)\times [\begin{array}{c} \frac{-l_{6}}{2}\\ 0\\ 0 \end{array}] \end{array}$$ (42)

$$X_{7}=[\begin{array}{c} q_{1}\\ q_{2}\\ 0 \end{array}]+Rot(q_{3},z)\times [\begin{array}{c} \frac{l_{1}}{2}+shoulderX\\ shoulderY\\ 0 \end{array}]+Rot(q_{3},z)\times Rot(q_{13},z)\times Rot(q_{14},x)\times Rot(q_{15},y)\times [\begin{array}{c} 0\\ \frac{l_{7}}{2}\\ 0 \end{array}]$$ (43)

$$X_{8}=X_{7}+Rot(q_{3},z)\times Rot(q_{13},z)\times Rot(q_{14},x)\times Rot(q_{15},y)\times (\left[\begin{array}{c} 0\\ \frac{l_{7}}{2}\\ 0 \end{array}]+Rot(q_{16},z)\times [\begin{array}{c} \frac{-l_{8}}{2}\\ 0\\ 0 \end{array}\right])$$ (44)

$$\begin{array}{ll} X_{9} & =X_{8}+Rot(q_{3},z)\times Rot(q_{13},z)\times Rot(q_{14},x)\times Rot(q_{15},y)\times Rot(q_{16},z)\times [\begin{array}{c} \frac{-l_{8}}{2}\\ 0\\ 0 \end{array}]\\ & +Rot(q_{17},z)\times Rot(q_{18},x)\times Rot(q_{19},y)\times [\begin{array}{c} \frac{-l_{9}}{2}\\ 0\\ 0 \end{array}] \end{array}$$ (45)

$$\begin{array}{ll} X_{10} & =[\begin{array}{c} q_{1}\\ q_{2}\\ 0 \end{array}]+Rot(q_{3},z)\times [\begin{array}{c} l_{1}\\ 0\\ 0 \end{array}]+Rot(q_{3},z)\times Rot(q_{4},z)\times [\begin{array}{c} \frac{l_{2}}{2}+pelvicX\\ -pelvicY\\ 0 \end{array}]\\ & +Rot(q_{3},z)\times Rot(q_{4},z)\times Rot(q_{20},z)\times Rot(q_{21},x)\times Rot(q_{22},y)\times [\begin{array}{c} 0\\ \frac{-l_{10}}{2}\\ 0 \end{array}] \end{array}$$ (46)

$$X_{11}=X_{10}+Rot(q_{3},z)\times Rot(q_{4},z)\times Rot(q_{20},z)\times Rot(q_{21},x)\times Rot(q_{22},y)\times (\left[\begin{array}{c} 0\\ \frac{-l_{10}}{2}\\ 0 \end{array}]+Rot(q_{23},z)\times [\begin{array}{c} \frac{l_{11}}{2}\\ 0\\ 0 \end{array}\right])$$ (47)

$$\begin{array}{ll} X_{12} & =X_{11}+Rot(q_{3},z)\times Rot(q_{4},z)\times Rot(q_{20},z)\times Rot(q_{21},x)\times Rot(q_{22},y)\times Rot(q_{23},z)\times [\begin{array}{c} \frac{l_{11}}{2}\\ 0\\ 0 \end{array}]\\ & +Rot(q_{24},z)\times Rot(q_{25},x)\times Rot(q_{26},y)\times [\begin{array}{c} \frac{l_{12}}{2}\\ 0\\ 0 \end{array}] \end{array}$$ (48)

$$\begin{array}{ll} X_{13} & =[\begin{array}{c} q_{1}\\ q_{2}\\ 0 \end{array}]+Rot(q_{3},z)\times [\begin{array}{c} l_{1}\\ 0\\ 0 \end{array}]+Rot(q_{3},z)\times Rot(q_{4},z)\times [\begin{array}{c} \frac{l_{2}}{2}+pelvicX\\ pelvicY\\ 0 \end{array}]\\ & +Rot(q_{3},z)\times Rot(q_{4},z)\times Rot(q_{27},z)\times Rot(q_{28},x)\times Rot(q_{29},y)\times [\begin{array}{c} 0\\ \frac{l_{13}}{2}\\ 0 \end{array}] \end{array}$$ (49)

$$X_{14}=X_{13}+Rot(q_{3},z)\times Rot(q_{4},z)\times Rot(q_{27},z)\times Rot(q_{28},x)\times Rot(q_{29},y)\times (\left[\begin{array}{c} 0\\ \frac{l_{13}}{2}\\ 0 \end{array}]+Rot(q_{30},z)\times [\begin{array}{c} \frac{l_{14}}{2}\\ 0\\ 0 \end{array}\right])$$ (50)

$$\begin{array}{ll} X_{15} & =X_{14}+Rot(q_{3},z)\times Rot(q_{4},z)\times Rot(q_{27},z)\times Rot(q_{28},x)\times Rot(q_{29},y)\times Rot(q_{30},z)\times [\begin{array}{c} \frac{l_{14}}{2}\\ 0\\ 0 \end{array}]\\ & +Rot(q_{31},z)\times Rot(q_{32},x)\times Rot(q_{33},y)\times [\begin{array}{c} \frac{l_{15}}{2}\\ 0\\ 0 \end{array}] \end{array}$$ (51)

where *Rot*(*q*_*i*_, *axis*) means that {*i*^*th*^} joint rotates in the *axis* direction. *ShoulderX* and *shoulderY* are the distances from the center of the mass of body_1_. *PelvicX* and *pelvicY* are the distances in the x and y directions from the center of the mass of body_2_.

## Supporting information

S1 File (TXT)

S1 Video (WMV)
